# Teilhabeorientierte Psychotherapie bei arbeitsfähigen und arbeitsunfähigen Patienten mit psychischen Erkrankungen: Ergebnisse einer Befragung niedergelassener Psychotherapeuten

**DOI:** 10.1007/s00103-024-03899-1

**Published:** 2024-05-28

**Authors:** Beate Muschalla, Michael Linden

**Affiliations:** 1 Klinische Psychologie, Psychotherapie und Diagnostik, Technische Universität Braunschweig, Humboldtstraße 33, 38106 Braunschweig, Deutschland; 2 Psychosomatische Medizin, Forschungsgruppe Psychosomatische Rehabilitation, Charité Universitätsmedizin Berlin, Berlin, Deutschland

**Keywords:** Arbeitsfähigkeit, Sozialmedizin, Psychische Erkrankungen, Psychotherapie, Psychotherapeuten, Work ability, Social medicine, Mental disorders, Psychotherapy, Psychotherapist

## Abstract

**Hintergrund:**

Die Behandlung (chronischer) psychischer Erkrankungen zielt sowohl auf die Symptomreduktion als auch auf die Verbesserung der sozialen und beruflichen Teilhabe durch sozialmedizinische Behandlungen und Beratungen ab. Ziel der vorliegenden Studie war es, arbeitsfähige und arbeitsunfähige Psychotherapiepatienten im Hinblick auf Gemeinsamkeiten und Unterschiede des Patientenstatus und der Behandlungssituation zu vergleichen.

**Methoden:**

73 kognitive Verhaltenstherapeuten und 58 psychodynamische Psychotherapeuten wurden zu 188 bzw. 134 aktuellen Patientenbehandlungen befragt. Die Fallberichte bezogen sich auf Patienten, die im Durchschnitt 42 Jahre alt waren (65 % Frauen).

**Ergebnisse:**

Es gab keine Unterschiede zwischen Patienten ohne oder mit kurzer Krankschreibung (bis zu 6 Wochen, *n* = 156) und Patienten mit längerer Krankschreibung (7 Wochen oder mehr, *n* = 140) in Bezug auf grundlegende Merkmale der Behandlung (Nebenwirkungen, therapeutische Allianz). Patienten mit längerer Krankheitsdauer hatten schwerere Beeinträchtigungen der Leistungsfähigkeit und Teilhabe. Sie erhielten spezifischere, auf die Teilhabe am Arbeitsleben ausgerichtete Behandlungen, während allgemeine salutotherapeutische Aktivitäten (Sportverein, Beratung, Familienunterstützung) bei Patienten mit kürzerer oder längerer Krankheitsdauer in gleicher Weise durchgeführt wurden.

**Diskussion:**

Die Therapeuten wählten die Behandlungsoptionen indikationsbezogen aus: Bei Patienten mit Problemen der Teilhabe am Arbeitsleben wurden mehr arbeitsbezogene Behandlungen durchgeführt, während Behandlungen zur Verbesserung der allgemeinen psychischen Gesundheit unabhängig von spezifischen Problemen der Teilhabe am Arbeitsleben eingesetzt wurden.

## Abstract (en)

**Background:**

Treatment of (chronic) mental disorders must focus on both reducing symptoms and improving social and work participation by social medicine treatments and counselling. The objective of this study was to compare psychotherapy patients who are fit or unfit for work to describe similarities and differences regarding patient status and interventions.

**Methods:**

Interviews were performed with 73 cognitive behavior therapists and 58 psychodynamic psychotherapists about 188 and 134 recent cases they had seen, respectively. The case reports referred to patients who were on average 42 years old (65% females).

**Results:**

There were no differences between patients with no or short-term sick leave (up to 6 weeks, *n* = 156) and patients with longer sick leave (7 weeks or more, *n* = 140) with respect to basic characteristics of treatment (side effects, therapeutic alliance). Patients with a longer sick leave duration had more severe capacity and participation impairments and received more specific work participation-oriented treatments, whereas general saluto-therapeutic activities (sports-club, counselling, family-support) were similarly undertaken in patients with shorter or longer sick leave.

**Discussion:**

Therapists chose intervention options according to indication: in patients with work participation problems, more work-related treatments are undertaken, whereas interventions for general mental health improvement are distributed independent of specific work participation problems.

## Hintergrund

Psychische Erkrankungen sind in der Regel chronisch [1–3] und gehen mit Teilhabeproblemen in verschiedenen Lebensbereichen einher, wobei insbesondere der Arbeitsplatz betroffen ist [4–7]. Hier sind psychische Erkrankungen häufig mit (Langzeit‑)Krankschreibungen [6–8] oder sogar Frühberentungen [4] verbunden. Patienten mit psychischen Erkrankungen sind jünger als Patienten mit somatischen Erkrankungen, wenn sie im Alter von etwa 50 Jahren in (Erwerbsminderungs‑)Rente gehen, und nur 50 % durchlaufen eine stationäre multiprofessionelle Rehabilitationsbehandlung, bevor sie eine Erwerbsminderungsrente zugesprochen bekommen [9, 10]. Psychische Erkrankungen sind in Deutschland der häufigste Grund für eine Erwerbsminderungsrente: 37–49 % aller neuen Erwerbsminderungsrenten pro Jahr sind auf psychische Erkrankungen zurückzuführen [11].

Während die epidemiologischen Häufigkeiten psychischer Erkrankungen in der Allgemeinbevölkerung als solche nicht zunehmen, sondern konstant bei etwa 30 % liegen [12], wachsen die Probleme im Arbeitsleben psychisch Erkrankter aufgrund veränderter Anforderungen und einer Verringerung von Freiheitsgraden für Abweichungen, die zum Verlust der Passung zwischen persönlichen Fähigkeiten und Arbeitsplatz führen [13, 14]. Die heutige Arbeitswelt stellt immer höhere psychosoziale Anforderungen, engeres Leistungs- und Zeitmonitoring, was insbesondere bei Mitarbeitern mit psychischen Erkrankungen zu Problemen führen kann, wenn diese ohnehin hinsichtlich Interaktion oder Belastbarkeit Probleme haben.

Ambulante Psychotherapeuten sind – ähnlich wie Hausärzte oder auch Therapeuten in Rehabilitationskliniken – daher nicht nur damit beschäftigt, die Symptome der akuten Krankheitsepisode ihrer chronisch psychisch kranken Patienten zu lindern; sie müssen sich auch mit deren Teilhabe an Arbeit und Leben befassen. Die Behandlungsziele bei chronischen Erkrankungen sollten sich daher an den verschiedenen Ebenen orientieren, die in der Internationalen Klassifikation der Funktionsfähigkeit, Behinderung und Gesundheit [15] vorgeschlagen werden. Eine alleinige Fokussierung auf Symptomreduktion ist unzureichend. Stattdessen ist eine mehrdimensionale und interdisziplinäre Behandlung erforderlich, die Symptomatik, Fähigkeiten und Kontext umfasst:

Eine *symptomorientierte Behandlung* – z. B. eine Exposition zur Verringerung von Panikattacken – kann als grundlegender Schritt einer ganzheitlichen Behandlung angesehen werden [15]. Da eine vollständige oder dauerhafte Symptomremission nur selten erreicht werden kann, ist das nächste Ziel die Verbesserung der durch die psychische Erkrankung beeinträchtigten Fähigkeiten, z. B. durch Psychoedukation, Verhaltenstraining und Beratung.

Zusätzlich kann eine *kontextorientierte Behandlung* durchgeführt werden, bei der das Umfeld angepasst wird, um die Teilhabe an Arbeit und Leben zu verbessern, z. B. durch eine stufenweise Wiedereingliederung am Arbeitsplatz (z. B. bei noch aufzutrainierender Ausdauerbelastbarkeit oder diesbezüglichen Insuffizienzängsten), eine qualitative Veränderung der Arbeit (z. B. Reduktion des Kundenkontakts bei Mitarbeitern mit sozialen Interaktionsschwierigkeiten) oder durch Unterstützung der Familie (z. B. mittels Feedback und Erinnerungen an bewährte Bewältigungsstrategien; [15, 16]).

Bislang gibt es keine Daten zu den Charakteristika von Psychotherapiepatienten mit unterschiedlich ausgeprägter Arbeitsfähigkeitsbeeinträchtigung sowie den Häufigkeiten und Arten fähigkeits- und kontextorientierter Behandlungen. In der vorliegenden Studie wird erstmals untersucht, ob und in welcher Weise es bei Patienten ohne Arbeitsunfähigkeit in den letzten 12 Monaten mit kurzfristiger und mit langfristiger Arbeitsunfähigkeit Unterschiede oder Gemeinsamkeiten gibt hinsichtlich:

1. ihres Krankheitsstatus, d. h. der Symptombelastung und der Einschränkungen der Leistungsfähigkeit und Teilhabe; und
2. der Behandlungen, die sie erhalten. Dabei wird das gesamte Spektrum krankheits- und kontextbezogener Behandlungen berücksichtigt, d. h. psychotherapeutische Methoden, Koordination mit Mitbehandlern, arbeitsorientierte Interventionen, Beratung und Interventionen zur Teilhabe am allgemeinen sozialen Leben.

## Methoden

### Auswahl der Psychotherapeuten

Die Untersuchung wurde in Berlin, Deutschland, im Rahmen eines größeren Projekts durchgeführt [17]. Die gesetzliche Krankenversicherung ermöglicht allen Versicherten bei Indikation psychotherapeutische Leistungen und erstattet bis zu 80 Sitzungen kognitiver Verhaltenstherapie (KVT) oder 120 Sitzungen psychodynamischer Psychotherapie (PDT). Psychotherapie wird von niedergelassenen Ärzten und Psychologen angeboten, die eine staatlich zugelassene spezifische Ausbildung in PDT oder KVT haben und verpflichtet sind, nur die Behandlung anzubieten, die sie beherrschen. Die hier vorliegende Untersuchung wurde mit psychologischen Psychotherapeuten durchgeführt, die nur Psychotherapie durchführen. In Berlin gibt es insgesamt 2051 psychologische Psychotherapeuten, das entspricht etwa einem pro 2000 Einwohner.

Unter Bezugnahme auf die offiziellen Listen wurden Psychotherapeuten kontaktiert, wobei keine spezielle Auswahl getroffen, sondern eine gleiche Anzahl von PDT- und KVT-Therapeuten angestrebt wurde. Die Therapeuten wurden über die Forschungsfragen informiert: Welche Charakteristika haben die Patienten, die den Weg zur Psychotherapie gefunden haben, und welche Behandlungen erhalten sie? Die Therapeuten gaben ihr Geschlecht, ihr Alter, ihre Ausbildung, ihre Praxisjahre, die Anzahl der Patienten und das vorherrschende Spektrum an behandelten Erkrankungen in ihrer Praxis an [17].

### Psychotherapeuteninterviews

131 ambulante Psychotherapeuten erklärten sich zur Teilnahme bereit und wurden von den Projektwissenschaftlern, die selbst Psychotherapeuten waren, in ihrer Praxis befragt. Die befragten Psychotherapeuten wurden gebeten, anonym die zuletzt gesehenen Patienten im arbeitsfähigen Alter zu nennen, die sie in mindestens 10 Therapiesitzungen behandelt hatten. Mittels dieser standardisierten und aufgrund des objektiven Kriteriums annähernd zufälligen Auswahl wurden 2 oder 3 Patienten pro Psychotherapeut beschrieben. Für jeden Patienten berichteten die Therapeuten soziodemografische Daten, therapiebezogene Angaben (Anzahl der Sitzungen, Qualität der therapeutischen Arbeitsbeziehung) und arbeitsbezogene Fakten (Dauer der Arbeitsunfähigkeit in den letzten 12 Monaten; [17]). Kerninhalt des Interviews waren im Folgenden krankheits- und behandlungsbezogene Aspekte, die anhand der folgenden Skalen bewertet wurden:

*Funktionsstörungen (Symptome)* des Patienten wurden mit der Visual Analogue Mood Scale (VAS; [18]) erfasst mit Bewertungen von negativ (0) bis gut (10) für die Symptombereiche Depressivität, Angst, Nervosität, somatoforme Symptome, Müdigkeit und Erschöpfung, Gedächtnisprobleme und wahnhafte Symptome.

Der *Schweregrad der Erkrankung* des Patienten wurde auf der Clinical-Global-Impression-Skala [18] von normal, überhaupt nicht krank [1] bis zu extrem kranken Patienten [7] bewertet.

Die arbeitsbezogenen *Fähigkeitsbeeinträchtigungen* des Patienten wurden mittels Mini-ICF-APP^1^ [19, 20] exploriert und im Fremdrating durch den Untersucher eingeschätzt. In Anlehnung an die Internationale Klassifikation der Funktionsfähigkeit, Behinderung und Gesundheit (ICF; [15]) werden im Mini-ICF-APP 13 Fähigkeiten exploriert, die typischerweise durch psychische Erkrankungen beeinträchtigt werden, nämlich: (1) Anpassung an Regeln und Routinen, (2) Planung und Strukturierung von Aufgaben, (3) Flexibilität, (4) Kompetenz- und Wissensanwendung, (5) Entscheidungs- und Urteilsfähigkeit, (6) Proaktivität, (7) Durchhaltefähigkeit, (8) Selbstbehauptung, (9) Kontaktfähigkeit zu Dritten, (10) Gruppenfähigkeit, (11) dyadische Beziehungen, (12) Selbstpflege und Selbstversorgung und (13) Mobilität. Jede Fähigkeitsdimension wurde auf einer 5‑stufigen Likert-Skala bewertet, von keine Beeinträchtigung (0) und subjektive Beeinträchtigung (1) über beobachtbare Beeinträchtigung (2), unterstützungsbedürftige Beeinträchtigung (3) bis hin zur vollständigen Beeinträchtigung (4). Der Grad der Fähigkeitsbeeinträchtigung wurde in Bezug auf den bestehenden Arbeitsplatz oder – im Falle von Arbeitslosigkeit – den allgemeinen Arbeitsmarkt bewertet. Ein Mittelwert gibt einen Eindruck vom Ausmaß der Gesamtbeeinträchtigung.

*Partizipationsstörungen* des Patienten wurden mit dem Index zur Messung von Einschränkungen der Teilhabe (IMEP; [21, 22]) erfasst, der (1) Aktivitäten des täglichen Lebens, (2) Freizeitaktivitäten, (3) soziale Aktivitäten, (4) enge Beziehungen, (5) besondere Stressoren im Leben und (6) Arbeit umfasst. Die Beurteilung erfolgte auf einer visuellen Analogskala von 0 = keine Beeinträchtigung bis 10 = volle Beeinträchtigung, d. h., in diesem Lebensbereich ist keine Aktivität möglich.

*Soziomedizinische Behandlungen,* die mit dem jeweiligen Patienten durchgeführt wurden, wurden in Anlehnung an die TOPP-Checkliste [23] kodiert, die 38 Items auflistet, darunter Aspekte der Zusammenarbeit mit Mitbehandlern, Kontakte mit Institutionen, arbeitsbezogene Interventionen sowie soziale Unterstützung und Beratung. Die Therapeuten gaben an, ob sie diese Interventionen bereits durchgeführt, initiiert oder perspektivisch vorgesehen haben.

### Datenanalysen

Die Daten wurden mit IBM SPSS Statistics (Version 26) ausgewertet. Explorative Gruppenvergleiche wurden mit Varianzanalyse (ANOVA) für kontinuierliche Variablen und Chi^2^-Test für Häufigkeiten berechnet [24].

## Ergebnisse

In Berlin wurden auf der Homepage der Kassenärztlichen Vereinigung Berlin 2051 Psychologische Richtlinienpsychotherapeuten aufgelistet. Davon wurden 900 angerufen. Erreicht werden konnten 365 Therapeuten. Mit 131 zur Untersuchung einwilligenden Therapeuten konnten Interviews geführt werden. Es waren 74 % weibliche Therapeuten mit einem Durchschnittsalter von 54 Jahren (SD = 10,97; Range 32–74). Im Durchschnitt waren sie seit 16,8 Jahren als Psychotherapeuten tätig (SD = 10,70; Range 1–45). Von ihnen waren 43,5 % psychodynamische Therapeuten, 55,7 % kognitive Verhaltenstherapeuten und ein Therapeut war in beiden Verfahren ausgebildet. Bezogen auf die zuletzt gesehenen Patienten im arbeitsfähigen Alter berichteten die Therapeuten über 322 Fallvignetten (58,4 % KVT, 41,6 % PDT). Die Patienten waren typische ambulante Psychotherapiepatienten: Sie waren im Durchschnitt 42 Jahre alt, 66 % waren weiblich. Diagnostiziert wurden in 1,6 % der Fälle eine hirnorganische Störung (ICD 10 F00–F09), in 2,5 % ein Drogenmissbrauch (F10–F19, ICD-10), in 3,8 % eine schizophrene Störung (F20–F29), in 61,7 % eine affektive Störung (F30–F39), in 30,4 % eine Angst- oder Zwangsstörung (F40–F42), 24,4 % eine stressbedingte Störung (F43), 15,8 % eine somatoforme Störung (F45) und 16,5 % eine Persönlichkeitsstörung (F60–69). Die durchschnittliche Dauer der aktuellen Krankheitsepisode betrug 8,29 Jahre (SD = 9,1; Range 1–35).

### Krankheitsstatus bei Patienten mit kurzer oder langandauernder Arbeitsunfähigkeit

In Tab. 1 sind die Daten zum Krankheitsstatus der Patienten berichtet. 47 % der Patienten waren länger als 7 Wochen krankgeschrieben. Es gab keine Unterschiede zwischen Patienten ohne oder mit kurzer Arbeitsunfähigkeit (bis zu 6 Wochen, *n* = 156) und Patienten mit längerer Arbeitsunfähigkeit (7 Wochen oder mehr, *n* = 140) in Bezug auf die Dauer der aktuellen Krankheitsepisode und die Anzahl der bisherigen ambulanten Behandlungen. Auch bei der Symptombelastung (VAS) gab es keine signifikanten Unterschiede zwischen Patienten mit kurzer und langer Arbeitsunfähigkeit.

**Tab. 1:** Charakteristika der Patientengruppe „arbeitsfähig“ und der Patientengruppen mit verschieden langen (kumulierten) Arbeitsunfähigkeitsdauern innerhalb der letzten 12 Monate. Mittelwerte (Standardabweichungen) bzw. relative Häufigkeiten

Patienten- und Behandlungscharakteristika	Arbeitsfähig in den letzten 12 Monaten (*n* = 96) (F)	Arbeitsunfähig 1–6 Wochen in den letzten 12 Monaten (*n* = 60) (1)	Arbeitsunfähig 7–26 Wochen in den letzten 12 Monaten (*n* = 70) (2)	Arbeitsunfähig 27–52 Wochen in den letzten 12 Monaten (*n* = 70) (3)	ANOVA oder *X*^2^ *p* (Bonferroni-korrigiert)
*Soziodemografische Charakteristika*					
Alter	38,83 (11,246)	39,90 (11,506)	42,33 (10,857)	44,97 (9,470)	Overall 0,002 Fvs1 1,000, Fvs2 0,244, Fvs3 0,002 1vs2 1,000, 1vs3 0,049, 2vs3 0,896
Geschlecht weiblich	61,5 %	73,3 %	64,3 %	62,9 %	Overall 0,451
Migrationshintergrund	17,7 %	19,0 %	7,5 %	4,2 %	Overall 0,051
Partnerschaft oder verheiratet	43,8 %	58,4 %	41,4 %	45,7 %	Overall 0,013
*Höchste berufliche Qualifikation* Ohne Berufsabschluss Handwerksmeister Berufsausbildung Universitätsabschluss Andere	24,0 % 1,0 % 36,5 % 35,4 % 3,1 %	13,3 % 3,3 % 41,7 % 38,3 % 3,3 %	12,9 % 2,9 % 47,1 % 35,7 % 1,4 %	15,7 % 0,0 % 65,7 % 17,1 % 1,4 %	Overall 0,038
Vollzeit/Teilzeit erwerbstätig	54,2 %	76,7 %	64,3 %	50,0 %	Overall 0,008
Bedeutung der Arbeit für den Erkrankungsstatus (1 = Arbeit ist hilfreiche Ressource, 4 = Arbeit wirkt pathogen)	1,81 (1,020)	2,47 (1,184)	2,63 (1,128)	2,93 (1,079)	Overall 0,000 Fvs1 0,004, Fvs2 0,000, Fvs3 0,000 1vs2 1,000, 1vs3 0,192, 2vs3 0,897
*Arbeitsplatzprobleme durch* Fehlzeiten Konflikte Unterforderung Überforderung Arbeitsumgebung	6 % 31 % 11 % 37 % 16 %	20 % 40 % 18 % 40 % 25 %	28 % 41 % 10 % 62 % 25 %	23 % 48 % 0 % 79 % 26 %	0,002 0,188 0,009 0,000 0,361
*Krankheitsstatus*					
VAS Depressivität 0 = negativ bis 10 = gut	4,55 (2,261)	4,03 (2,201)	4,09 (2,138)	4,26 (2,320)	Overall 0,445 Fvs1 0,957, Fvs2 1,000, Fvs3 1,000 1vs2 1,000, 1vs3 1,000, 2vs3 1,000
VAS Angst 0 = negativ bis 10 = gut	4,25 (2,215)	3,70 (2,069)	3,60 (1,837)	3,56 (2,237)	Overall 0,112 Fvs1 0,683, Fvs2 0,304, Fvs3 0,224 1vs2 1,000, 1vs3 1,000, 2vs3 1,000
VAS Nervosität 0 = negativ bis 10 = gut	4,19 (2,089)	3,62 (1,878)	3,47 (2,118)	3,56 (2,282)	Overall 0,104 Fvs1 0,601, Fvs2 0,187, Fvs3 0,345 1vs2 1,000, 1vs3 1,000, 2vs3 1,000
VAS somatoforme Beschwerden 0 = negativ bis 10 = gut	5,97 (2,889)	4,88 (2,793)	5,01 (2,831)	4,53 (2,647)	Overall 0,007 Fvs1 0,601, Fvs2 0,187, Fvs3 0,345 1vs2 1,000, 1vs3 1,000, 2vs3 1,000
VAS Energieverlust 0 = negativ bis 10 = gut	5,57 (2,570)	4,82 (2,167)	4,31 (2,177)	4,20 (2,332)	Overall 0,001 Fvs1 0,115, Fvs2 0,185, Fvs3 0,007 1vs2 1,000, 1vs3 1,000, 2vs3 1,000
VAS Merkfähigkeitsprobleme 0 = negativ bis 10 = gut	7,46 (2,214)	6,67 (2,562)	6,31 (2,821)	5,77 (2,671)	Overall 0,000 Fvs1 0,360, Fvs2 0,027, Fvs3 0,000 1vs2 1,000, 1vs3 0,280, 2vs3 1,000
VAS Denkstörungen 0 = negativ bis 10 = gut	8,72 (2,126)	8,88 (1,776)	8,51 (2,314)	8,73 (2,180)	Overall 0,800 Fvs1 1,000, Fvs2 1,000, Fvs3 1,000 1vs2 1,000, 1vs3 1,000, 2vs3 1,000
VAS Mittelwert 0 = negativ bis 10 = gut	5,8155 (1,50101)	5,2286 (1,48769)	5,0449 (1,57745)	4,9429 (1,70276)	Overall 0,001 Fvs1 0,141, Fvs2 0,012, Fvs3 0,003 1vs2 1,000, 1vs3 1,000, 2vs3 1,000
Aktuelle Krankheitsschwere (CGI) 1 = gesund, 7 = schwerkrank	5,32 (1,18)	5,53 (1,17)	5,87 (1,00)	6,33 (0,83)	Overall 0,000 Fvs1 1,000, Fvs2 0,007, Fvs3 0,000 1vs2 0,433, 1vs3 0,000, 2vs3 0,070
Parallele Behandlung der psychischen Erkrankung durch Arzt	74,0 %	90,0 %	95,7 %	97,1 %	0,000
Arbeitsunfähigkeitsdauer in den letzten 12 Monaten in Wochen	0,00 (0,000)	3,52 (1,846)	14,36 (6,143)	47,69 (7,292)	Overall 0,000 Fvs1 0,000. Fvs2 0,000, Fvs3 0,000 1vs2 0,000, 1vs3 0,000, 2vs3 0,000
Dauer der aktuellen Krankheitsepisode in Wochen	50,4 (57,67)	45,40 (68,550)	31,90 (57,942)	46,23 (63,439)	Overall 0,066 Fvs1 1,000, Fvs2 0,347, Fvs3 1,000 1vs2 1,000, 1vs3 1,000, 2vs3 1,000
Anzahl ambulanter Psychotherapien bislang	0,84 (1,067)	0,58 (0,821)	0,59 (0,0873)	0,57 (0,789)	Overall 0,227 Fvs1 0,775, Fvs2 0,722, Fvs3 0,500 1vs2 1,000, 1vs3 1,000, 2vs3 1,000
Anzahl stationärer akuter Psychotherapien bislang	0,56 (1,264)	0,43 (0,939)	0,74 (1,539)	0,96 (2,579)	Overall 0,297 Fvs1 1,000, Fvs2 1,000, Fvs3 0,826 1vs2 1,000, 1vs3 0,483, 2vs3 1,000
Anzahl stationärer psychosomatischer Rehabilitationsbehandlungen bislang	0,21 (0,501)	0,35 (0,755)	0,36 (0,566)	0,77 (1,010)	Overall 0,000 Fvs1 1,000, Fvs2 1,000, Fvs3 0,000 1vs2 1,000, 1vs3 0,006, 2vs3 0,004
*Fähigkeitsbeeinträchtigungen und Partizipationsstörungen*					
Mini-ICF-APP Fähigkeitsbeeinträchtigungen global Mittelwert 0 = keine Beeinträchtigung, 4 = vollständige Beeinträchtigung	1,7356 (0,171378)	1,8579 (0,52597)	1,9725 (0,58098)	2,2703 (0,72678)	Overall 0,000 Fvs1 1,000, Fvs2 0,130, Fvs3 0,000 1vs2 1,000, 1vs3 0,002, 2vs3 0,044
IMEP Partizipationsstörung Mittelwert 0 = keine Beeinträchtigung, 10 = volle Beeinträchtigung	3,9253 (1,77340)	4,6389 (1,85368)	4,9179 (1,66927)	5,8190 (1,83207)	Overall 0,000 Fvs1 0,093, Fvs2 0,003, Fvs3 0,000 1vs2 1,000, 1vs3 0,001, 2vs3 0,019
IMEP Aktivitäten des täglichen Lebens 0 = keine Beeinträchtigung, 10 = volle Beeinträchtigung	2,35 (1,789)	3,00 (2,270)	3,43 (2,563)	4,30 (2,475)	Overall 0,000 Fvs1 0,497, Fvs2 0,016, Fvs3 0,000 1vs2 1,000, 1vs3 .007, 2vs3 0,137
IMEP Erholungsaktivitäten 0 = keine Beeinträchtigung, 10 = volle Beeinträchtigung	3,57 (2,116)	4,10 (2,334)	4,14 (2,135)	5,26 (2,558)	Overall 0,000 Fvs1 0,963, Fvs2 0,673, Fvs3 0,000 1vs2 1,000, 1vs3 0,025, 2vs3 0,024
IMEP soziale Aktivitäten 0 = keine Beeinträchtigung, 10 = volle Beeinträchtigung	3,82 (2,441)	4,45 (2,235)	4,58 (2,511)	5,56 (2,441)	Overall 0,000 Fvs1 0,697, Fvs2 0,290, Fvs3 0,000 1vs2 1,000, 1vs3 0,058, 2vs3 0,107
IMEP enge Beziehungen 0 = keine Beeinträchtigung, 10 = volle Beeinträchtigung	4,92 (3,056)	5,27 (2,957)	4,93 (2,815)	5,51 (3,234)	Overall 0,565 Fvs1 1,000, Fvs2 1,000, Fvs3 1,000 1vs2 1,000, 1vs3 1,000, 2vs3 1,000
IMEP Stressbewältigung 0 = keine Beeinträchtigung, 10 = volle Beeinträchtigung	5,14 (2,532)	5,92 (2,011)	6,21 (2,245)	7,10 (2,188)	Overall 0,000 Fvs1 0,232, Fvs2 0,017, Fvs3 0,000 1vs2 1,000, 1vs3 0,021, 2vs3 0,136
IMEP Arbeit und berufliche Aktivitäten 0 = keine Beeinträchtigung, 10 = volle Beeinträchtigung	3,75 (2,854)	5,10 (2,772)	6,23 (2,845)	7,19 (3,688)	Overall 0,000 Fvs1 0,046, Fvs2 0,000, Fvs3 0,000 1vs2 0,219, 1vs3 0,001, 2vs3 0,389
*Psychotherapieprozess*					
Wie viele Therapiestunden haben bislang stattgefunden?	47,73 (53.865)	36,37 (22.648)	41,61 (41.274)	43,14 (37.278)	Overall 0,430 Fvs1 0,616, Fvs2 1,000, Fvs3 1,000 1vs2 1,000, 1vs3 1,000, 2vs3 1,000
Gute Patient-Therapeut-Beziehung Rating 1–4	3,52 (0,542)	3,45 (0,565)	3,53 (0,531)	3,44 (0,605)	Overall 0,700 Fvs1 1,000, Fvs2 1,000, Fvs3 1,000 1vs2 1,000, 1vs3 1,000, 2vs3 1,000
Keine Behandlungsnebenwirkungen	41,7 %	25,0 %	31,4 %	27,1 %	0,103
Behandlungsprognose 1 = Remission 2 = Dauerbeeinträchtigung 3 = schwere Dauerbeeinträchtigung	1,82 (0,615)	1,92 (0,619)	1,89 (0,627)	2,17 (0,636)	Overall 0,004 Fvs1 1,000, Fvs2 1,000, Fvs3 0,003 1vs2 1,000, 1vs3 0,126, 2vs3 0,043
Arbeitsfähigkeit zum Behandlungsende 1 = Arbeitsfähig 2 = 3 = Arbeitsunfähig	1,25 (0,461)	1,38 (0,555)	1,47 (0,607)	1,84 (0,715)	Overall 0,000 Fvs1 1,000, Fvs2 0,107, Fvs3 0,000 1vs2 1,000, 1vs3 0,000, 2vs3 0,001
*Koordination mit Mitbehandlern*					
Facharzt	40,6 %	40,0 %	57,1 %	67,1 %	0,000
Stationäre psychosomatische Rehabilitation	10,4 %	25,0 %	30,0 %	40,0 %	0,000
Stationäre psychiatrische Behandlung	6,3 %	15,0 %	18,8 %	17,1 %	0,137
Selbsthilfegruppe	4,2 %	13,3 %	12,9 %	17,1 %	0,063
Psychoedukationsseminare	9,4 %	10,0 %	17,1 %	11,4 %	0,279
Ergotherapie	7,3 %	3,3 %	8,6 %	10,0 %	0,074
Sozialpsychiatrischer Dienst	6,3 %	3,3 %	8,6 %	7,1 %	0,215
Drogenberatung	2,1 %	5,0 %	4,3 %	10,0 %	0,332
Nachsorgegruppe nach stationärer Behandlung	1,0 %	1,7 %	1,4 %	8,6 %	0,000
Soziotherapie	0,0 %	0,0 %	7,1 %	2,9 %	0,002
Medizinischer Dienst der Krankenkasse (MDK)	9,4 %	11,7 %	34,3 %	35,7 %	0,000
Tagesklinik	1,0 %	1,7 %	2,9 %	7,1 %	0,012
Entzugsbehandlung	2,1 %	3,3 %	1,4 %	5,7 %	0,762
Ambulanter Pflegedienst	3,1 %	3,3 %	4,3 %	2,9 %	0,832
*Arbeit und berufliche Teilhabe*					
Arbeitsunfähigkeitsbescheinigung	5,2 %	31,7 %	44,3 %	45,7 %	0,000
Jobcenter	15,6 %	5,0 %	18,6 %	28,6 %	0,005
Stufenweise Wiedereingliederung	4,2 %	5,0 %	30,0 %	17,1 %	0,000
Erwerbsminderungsrente	2,1 %	3,3 %	4,3 %	15,7 %	0,000
Rehaberatung	8,3 %	5,0 %	11,4 %	22,9 %	0,000
Berufliche Rehabilitation (LTA)	7,3 %	0,0 %	11,4 %	5,7 %	0,000
Betriebliches Eingliederungsmanagement	1,0 %	1,7 %	17,1 %	12,9 %	0,000
Betriebsrat	3,1 %	6,7 %	7,1 %	8,6 %	0,007
Betriebsarzt	2,1 %	5,0 %	20,0 %	15,7 %	0,002
*Soziale Teilhabe*					
Sportverein	22,9 %	28,3 %	30,0 %	25,7 %	0,366
Familienkontakte	5,2 %	10,0 %	12,9 %	7,1 %	0,098
Familienberatung	7,3 %	6,7 %	10,0 %	5,7 %	0,291
Beratungsstelle	5,2 %	3,3 %	5,7 %	8,6 %	0,462
Familienhilfe	6,3 %	5,0 %	5,7 %	2,9 %	0,804
Betreuer	2,1 %	1,7 %	2,9 %	8,6 %	0,102
Drogenberatung	2,1 %	1,7 %	5,7 %	4,3 %	0,820
Geschütztes Wohnen	3,1 %	3,3 %	4,3 %	2,9 %	0,832
Einzelfallhilfe	2,1 %	3,3 %	4,3 %	0,0 %	0,098
Psychiatrische Hauskrankenpflege	0,0 %	0,0 %	4,3 %	1,4 %	0,183
Integrationsamt	3,1 %	0,0 %	1,4 %	1,4 %	0,091
Hausbesuche durch einen Therapeuten	2,1 %	0,0 %	1,4 %	1,4 %	0,795

*CGI* Clinical Global Impression Scale, *IMEP* Index zur Messung von Einschränkungen der Teilhabe Fremdratingversion, *LTA* Leistungen zur Teilhabe am Arbeitsleben, *Mini-ICF-APP* Kurzinstrument zur Fremdbeurteilung von Aktivitäts- und Partizipationsstörungen bei psychischen Erkrankungen, *VAS* visuelle Analogskala der psychischen Symptombelastung, *Fvs1* Vergleichspaar entsprechend Tabellenkopf, hier z. B. arbeitsfähige Patienten (F) versus arbeitsunfähige Patienten mit 1–6 Wochen in den letzten 12 Monaten (1)

Patienten mit längerer Krankschreibung waren jedoch signifikant stärker in ihren Fähigkeiten (Mini-ICF-APP; [19, 20]) und ihren täglichen und sozialen Aktivitäten, der Stressbewältigung und der Teilnahme am Arbeitsleben beeinträchtigt [21, 22].

### Behandlungsstatus bei Patienten mit kurzer oder langandauernder Arbeitsunfähigkeit

In Tab. 1 sind die Daten zum Behandlungsstatus der Patienten berichtet. Die Qualität der Zusammenarbeit zwischen Patient und Therapeut war in allen 3 Gruppen ähnlich, unabhängig vom Ausmaß der Arbeitsunfähigkeitsproblematik. Auch Nebenwirkungen traten in ähnlicher Häufigkeit auf. Die Therapeuten sahen die Prognose bei Patienten, die länger als ein halbes Jahr arbeitsunfähig waren, deutlich schlechter als bei den anderen Patienten.

Es gab mehr zusätzliche Therapien bei Patienten, die länger arbeitsunfähig waren (Koordination mit Mitbehandlern), jedoch wurden edukative Interventionen und Beratungsgespräche (psychoedukative Seminare, Drogenberatung, sozialpsychiatrischer Dienst) mit ähnlicher Häufigkeit bei Patienten ohne oder mit Langzeitarbeitsunfähigkeit durchgeführt.

Fähigkeits- und kontextorientierte salutotherapeutische Aktivitäten, die auf die Teilhabe am allgemeinen Leben abzielen (z. B. Sportverein, Beratung, Unterstützung der Familie), wurden bei Patienten mit kürzerer oder längerer Arbeitsunfähigkeit in ähnlicher Weise durchgeführt.

Patienten mit einer längeren Arbeitsunfähigkeitsdauer erhielten spezifischere, auf die Teilhabe am Arbeitsleben ausgerichtete kontextbezogene Behandlungen (z. B. Arbeitsunfähigkeit, stufenweise Wiedereingliederung, stationäre Rehabilitationsberatung, Koordination mit dem Betriebsarzt).

## Diskussion

Die Daten zeigen, dass Psychotherapeuten regelmäßig Patienten mit Arbeitsfähigkeitsbeeinträchtigungen behandeln: 47 % der Patienten waren länger als 7 Wochen krankgeschrieben. Wie aus der Literatur bekannt ist [1–3, 6], sind psychische Probleme in der Regel chronisch: Selbst aktuelle Krankheitsepisoden können mehrere Jahre andauern, die Patienten werden häufig parallel behandelt und sie sind bei Antritt einer Psychotherapie auch bereits vorbehandelt. Patienten, die länger „krankgeschrieben“ (arbeitsunfähig) sind, sind stärker beeinträchtigt, was ein Hinweis dafür ist, dass Arbeitsunfähigkeiten valide sind und ihre Berechtigung haben.

Entsprechend diesen Merkmalen ihrer Patienten müssen sich Psychotherapeuten um arbeitsbezogene Teilhabebeeinträchtigungen kümmern, wobei sie gemäß dem multimodalen ICF-Modell [14] unterschiedliche Interventionsmethoden anwenden: Es werden symptomorientierte Behandlungen eingesetzt, aber auch fähigkeits- und kontextorientierte Behandlungen im Sinne einer Unterstützung der Arbeit und der allgemeinen Teilhabe am gesellschaftlichen Leben. Das Spektrum der von den Psychotherapeuten genannten Behandlungsaktivitäten ist vergleichbar mit Behandlungsinitiativen, die von Hausärzten in der Routineversorgung von Patienten mit psychischen Erkrankungen durchgeführt werden [22, 23].

Limitationen bestehen in der Selektivität der Stichprobe: Da die Teilnahme freiwillig war, ist davon auszugehen, dass ggf. besonders interessierte, problembewusste und engagierte Kollegen an der Untersuchung mitgewirkt haben. Immerhin hat von den erreichten ein gutes Drittel an der Untersuchung teilgenommen, was auf ein hohes Interesse und Einsatzbereitschaft der Psychotherapeuten hinweist. Die Daten zeigen also, was an teilhabeorientierter Behandlung in ambulanter psychotherapeutischer Praxis bei guten Voraussetzungen seitens der Psychotherapeuten möglich ist. Die Daten sind nicht repräsentativ für alle Psychotherapeuten, jedoch prototypisch repräsentativ, im Sinne grundsätzlicher Machbarkeit sozialmedizinisch und teilhabeorientierter Behandlung. Durch die strukturierte Abfrage der Patienten- und Behandlungscharakteristika zu den Fällen sollte Erinnerungsverfälschungen oder Antworttendenzen in den Therapeuteninterviews entgegengewirkt worden sein.

Insgesamt zeigen die Daten, dass Psychotherapeuten in der Lage sind, Chronizität und unterschiedliche Beeinträchtigungsgrade zu erkennen und dementsprechend gezielt Behandlungen auszuwählen: Bei langzeitarbeitsunfähigen Patienten wurden mehr arbeitsbezogene Interventionen durchgeführt, während Behandlungen, die eher die allgemeine Teilhabe und salutogene Ziele (Gesundheitsverhalten fördern) betreffen, gleichmäßig auf die 4 Patientengruppen verteilt waren. Die Ergebnisse stützen somit die Annahme, dass je nach Indikation unterschiedliche Behandlungsformen zum Einsatz gebracht werden:

1. symptomorientierte Behandlungen bzw. medizinische Behandlungen,
2. salutogenetische, allgemein gesundheitsfördernde Behandlungen und Beratungen und
3. spezifische beeinträchtigungsorientierte sozialmedizinische Behandlungen, wie z. B. Maßnahmen zur beruflichen Eingliederung.

## Fazit

Die Daten dieser Untersuchung zeigen, dass Psychotherapeuten chronische Beeinträchtigungen aufgrund psychischer Erkrankungen und die Notwendigkeit sozialmedizinischer Interventionen verstehen und – ähnlich wie Ärzte [23] – diese Interventionen entsprechend den Bedürfnissen der verschiedenen Patientengruppen handhaben. Dazu gehört auch die Integration einer kurzen Psychotherapie von 12–45 Sitzungen in die lang andauernde allgemeinmedizinische Behandlung durch einen Hausarzt: 40–67 % der Patienten hatten eine parallele Behandlung durch einen Hausarzt (neben der psychotherapeutischen Behandlung mit begrenzter Anzahl von Sitzungen).

Die Anwendung verschiedener Behandlungsmethoden sollte der Goldstandard für die Verbesserung des langfristigen biopsychosozialen Gesundheitszustands [15] von Patienten mit chronischen psychischen Erkrankungen sein, wobei nicht nur die akute Krankheitsepisode zu berücksichtigen ist, sondern auch die Frage zu stellen ist: Wie kann der Patient mit seiner Krankheit gut umgehen und trotz wiederkehrender Symptomepisoden in den nächsten 20, 30, 40 Jahren seines Lebens erfolgreich am Arbeits- und Sozialleben teilnehmen?

In der Psychotherapeutenaus- und -weiterbildung sollten sozialmedizinische und teilhabeorientierte Behandlungsaspekte und die Lebensspanneorientierung als therapeutisch handlungsleitend vermittelt werden.

Im Hinblick auf die Versorgungsforschung könnte von Interesse sein, wie die neuen Verordnungsbefugnisse (Ergotherapie, Soziotherapie, häusliche Krankenpflege) durch Psychotherapeuten Nutzung erfahren.

## Data Availability

Daten und Materialien sind auf Anfrage bei den Autoren erhältlich.

## Footnotes

1 Mini-ICF-APP: Kurzinstrument zur Fremdbeurteilung von Aktivitäts- und Partizipationsstörungen bei psychischen Erkrankungen in Anlehnung an die Internationale Klassifikation der Funktionsfähigkeit, Behinderung und Gesundheit (ICF) der Weltgesundheitsorganisation; APP: Assessment of Activities and Participation in Psychological Disorders.

## References

[1] Gilbert P, Allan S, Nicholls W, Olsen K (2005). The assessment of psychological symptoms of patients referred to community mental health teams: distress, chronicity and life interference. Clin Psychol Psychother.

[2] Kühn K, Quednow B, Barkow K, Heun R, Linden M, Maier W (2002). Chronifizierung und psychosoziale Behinderung durch depressive Erkrankungen bei Patienten in der Allgemeinarztpraxis im Einjahresverlauf Ergebnisse aus einer Studie der Weltgesundheitsorganisation. Nervenarzt.

[3] Möller HJ, Laux G, Deister A, Braun-Scharm H (2005). Psychiatrie und Psychotherapie.

[4] Linden M (2017). Definition and assessment of disability in mental disorders under the perspective of the International Classification of Functioning disability and health (ICF). Behav Sci Law.

[5] Muschalla B, Vilain M, Lawall C, Lewerenz M, Linden M (2012). Participation restrictions at work indicate participation restrictions in other domains of life. Psychol Health Med.

[6] Stansfeld S, Feeney A, Head J, Canner R, North F, Marmot M (1995). Sickness absence for psychiatric illness: the Whitehall II Study. Soc Sci Med.

[7] Stansfeld SA, Clark C, Caldwell T, Rodgers B, Power C (2008). Psychosocial work characteristics and anxiety and depressive disorders in midlife: the effects of prior psychological distress. Occup Environ Med.

[8] Doran CM, Kinchin I (2017). A review of the economic impact of mental illness. Aus: Health Rev.

[9] Blomgren J, Perhoniemi R (2021). Increase in sickness absence due to mental disorders in Finland: trends by gender, age and diagnostic group in 2005–2019.

[10] Roski C, Romppel M, Grande G (2015). Risk factors for disability pensioning caused by mental disorders—a systematic review. Gesundheitswesen.

[11] DRV (2020) Rentenversicherung in Zahlen 2020. https://www.sozialpolitik-aktuell.de/files/sozialpolitik-aktuell/_Politikfelder/Alter-Rente/Dokumente/2020_10_DRV_Rentenvers_in_Zahlen.pdf. Zugegriffen: 16. Jan. 2024

[12] Wittchen HU, Jacobi F, Rehm J, Gustavsson A, Svensson M, Jönsson B, Olesen J, Allgulander C, Alonso J, Faravelli C (2011). The size and burden of mental disorders and other disorders of the brain in Europe 2010. Eur Neuropsychopharmacol.

[13] Edwards JR, Van Harrison R (1993). Job demands and worker health: three-dimensional reexamination of the relationship between person-environment fit and strain. J Appl Psychol.

[14] French Jr JR (1973). Person role fit. Occup Ment Health.

[15] WHO (2001). International Classification of Functioning, disability and health (ICF).

[16] United Nations (2006) United Nations conventions on the rights of persons with disabilities. https://www.un.org/disabilities/documents/convention/convention_accessible_pdf.pdf. Zugegriffen: 16. Jan. 2024

[17] Muschalla B, Linden M, Rose M (2021). Patients’ characteristics and psychosocial treatment in psychodynamic and cognitive behavior therapy. Front Psychiatry.

[18] Guy W (1976) Clinical global impression. Assess Man Psychopharmacol: 217–222

[19] Linden M, Baron S, Muschalla B (2009). Mini-ICF-PP-Rating für psychische Störungen (Mini-ICF-APP). Ein Kurzinstrument zur Beurteilung von Fähigkeits- bzw. Partizipationsstörungen bei Psychischen Störungen.

[20] AWMF (2019) Leitlinie zur Begutachtung psychischer und psychosomatischer Störungen. https://register.awmf.org/assets/guidelines/051-029l_S2k_Begutachtung-psychischer-psychosomatischer-Stoerungen_2019-12_01.pdf. Zugegriffen: 16. Jan. 2024 (Arbeitsgemeinschaft der Wissenschaftlichen Medizinischen Fachgesellschaften AWMF)

[21] Deck R (2007). Veränderungen von Teilhabestörungen nach Reha. Prax Klin Verhaltensmed Rehabil.

[22] Linden M, Deck R, Muschalla B (2018). Rate and spectrum of participation impairment in patients with chronic mental disorders: comparison of self-and expert ratings. Contemp Behav Health Care.

[23] Linden M, Muschalla B, Noack N, Heintze C, Doepfmer S (2018) Treatment changes in general practice patients with chronic mental disorders following a psychiatric–psychosomatic consultation. Health Serv Res Manage Epidemiol 5: (2333392818758523.) 10.1177/2333392818758523 PMC5858609 29568790

[24] Field A (2018). Discovering Statistics Using SPSS.

